# Follow-up SARS-CoV-2 serological study of a health care worker cohort following COVID-19 booster vaccination

**DOI:** 10.1186/s12879-024-09338-5

**Published:** 2024-04-24

**Authors:** Alexander Hönning, Sara Tomczyk, Julia Hermes, Marica Grossegesse, Natalie Hofmann, Janine Michel, Markus Neumann, Andreas Nitsche, Berthold Hoppe, Tim Eckmanns, Hajo Schmidt-Traub, Kristina Zappel

**Affiliations:** 1 Centre for Clinical Research, BG Klinikum Unfallkrankenhaus Berlin gGmbH, Berlin, Germany; 2 Department of Infectious Disease Epidemiology, Robert Koch Institute, Berlin, Germany; 3 Highly Pathogenic Viruses, Centre for Biological Threats and Special Pathogens, WHO Reference Laboratory for SARS-CoV-2 and WHO Collaborating Centre for Emerging Infections and Biological Threats, Robert Koch Institute, Berlin, Germany; 4 Health and Medical University Potsdam, Potsdam, Germany; 5 Institute of Laboratory Medicine, BG Klinikum Unfallkrankenhaus Berlin gGmbH, Berlin, Germany; 6 BG Klinikum Unfallkrankenhaus Berlin gGmbH, Berlin, Germany

**Keywords:** SARS-CoV-2, Vaccine, Health care worker, Tertiary care hospital, Germany

## Abstract

**Background:**

Studies have shown that Omicron breakthrough infections can occur at higher SARS-CoV-2 antibody levels compared to previous variants. Estimating the magnitude of immunological protection induced from COVID-19 vaccination and previous infection remains important due to varying local pandemic dynamics and types of vaccination programmes, particularly among at-risk populations such as health care workers (HCWs). We analysed a follow-up SARS-CoV-2 serological survey of HCWs at a tertiary COVID-19 referral hospital in Germany following the onset of the Omicron variant.

**Methods:**

The serological survey was conducted in January 2022, one year after previous surveys in 2020 and the availability of COVID-19 boosters including BNT162b2, ChAdOx1-S, and mRNA-1273. HCWs voluntarily provided blood for serology and completed a comprehensive questionnaire. SARS-CoV-2 serological analyses were performed using an Immunoglobulin G (IgG) enzyme-linked immunosorbent assay (ELISA). Antibody levels were reported according to HCW demographic and occupational characteristics, COVID-19 vaccination and SARS-CoV-2 infection history, and multivariate linear regression was used to evaluate these associations.

**Results:**

In January 2022 (following the fourth COVID-19 wave in Germany including the onset of the Omicron variant), 1482/1517 (97.7%) HCWs tested SARS-CoV-2 seropositive, compared to 4.6% in December 2020 (second COVID-19 wave). Approximately 80% had received three COVID-19 vaccine doses and 15% reported a previous laboratory-confirmed SARS-CoV-2 infection. SARS-CoV-2 IgG geometric mean titres ranged from 335 (95% Confidence Intervals [CI]: 258–434) among those vaccinated twice and without previous infection to 2204 (95% CI: 1919–2531) among those vaccinated three times and with previous infection. Heterologous COVID-19 vaccination combinations including a mRNA-1273 booster were significantly associated with the highest IgG antibody levels compared to other schemes. There was an 8-to 10-fold increase in IgG antibody levels among 31 HCWs who reported a SARS-CoV-2 infection in May 2020 to January 2022 after COVID-19 booster vaccination.

**Conclusions:**

Our findings demonstrate the importance of ongoing COVID-19 booster vaccination strategies in the context of variants such as Omicron and despite hybrid immunity from previous SARS-CoV-2 infections, particularly for at-risk populations such as HCWs. Where feasible, effective types of booster vaccination, such as mRNA vaccines, and the appropriate timing of administration should be carefully considered.

**Supplementary Information:**

The online version contains supplementary material available at 10.1186/s12879-024-09338-5.

## Background

In January 2022, the Omicron variant (B.1.1.529) of SARS-CoV-2 had been identified in all European Union/European Economic Area (EU/EEA) countries and was a globally dominant strain [1]. Although the Omicron variant and its subsequent subvariants were later found to be associated with a lower risk of death and hospitalization compared to the previously dominant Delta variant [2, 3], concerns for ongoing breakthrough infections remained due to Omicron’s increased transmissibility and resistance to neutralization by vaccine-induced antibodies [4, 5]. As of January 2024, Omicron lineages XBB.1.5, XBB.1.16, EG.5, BA.2.86 and JN.1 continue to be global circulating variants of interest from the World Health Organisation (WHO) [6].

Despite the continuously evolving dynamics of the COVID-19 pandemic, health care workers (HCWs) remain an important group-of-interest for investigation. A systematic review including studies from 2021 to 2022 found that breakthrough infections in individuals who had completed a primary COVID-19 vaccination series were more common in HCWs [7]. Further HCW studies showed that Omicron breakthrough infections occurred at higher anti-RBD-IgG serum levels at the time of diagnosis compared to those of previous variants [8–10]. In this context, evidence-based infection prevention and control measures such as COVID-19 vaccination recommendations are particularly warranted for HCWs with high-risk occupational exposure to SARS-CoV-2 and a potential role in further nosocomial transmission.

Initial studies investigating the primary COVID-19 vaccine series among HCWs showed that the level of SARS-CoV-2 Immunoglobulin (IgG) antibodies often decreased at a consistent rate in the six months following the receipt of the second dose and at higher levels among those with prior SARS-CoV-2 infection [11, 12]. Studies then highlighted the role of booster vaccination to increase SARS-CoV-2 antibody responses, including evidence demonstrating high IgG antibody responses with heterologous immunization combining inactivated and mRNA vaccines [13]. Most recently, a systematic review showed that hybrid immunity developed through both SARS-CoV-2 infection and vaccination had the highest protective effectiveness [14]. This review of 15 studies found only one study among healthcare workers. Estimating the magnitude of immunological protection induced from vaccination and previous infection remains challenging due to varying local pandemic dynamics and vaccination programmes including different types of vaccinations and number of doses.

In order to continue to add to this body of evidence, we analysed a follow-up serological survey of HCWs in 2022 at a tertiary COVID-19 referral hospital, one year after previous survey in May-June and December 2020. The aim was to assess the magnitude of SARS-CoV-2 antibody levels in the context of varying HCW characteristics, SARS-CoV-2 infection history, and different COVID-19 vaccination schemes as well as to describe the antibody kinetics over time among the subset participating in all surveys.

## Methods

### Study site

The tertiary care hospital ‘Unfallkrankenhaus Berlin (ukb)’ in Berlin, Germany, is a maximum care trauma centre with more than 730 beds and over 2,500 HCWs serving a catchment area of approximately 300,000 inhabitants. According to the ‘SAVE-Model’ in Berlin (i.e. distribution of COVID-19 patients requiring invasive ventilation), the ukb hospital served as one of the 16 specialized hospitals with the capacity to treat critically-ill COVID-19 patients [15]. Since 2020, they have implemented a comprehensive set of SARS-CoV-2 prevention and control protocols as previously described [16].

### Study design

Following the ‘first wave’ of the COVID-19 outbreak in Germany in May-June 2020, a longitudinal seroepidemiological investigation among the HCWs at this tertiary hospital (including those with and without direct patient contact) was conducted, as previously published [16, 17]. In short, this included two timepoints in May/June (*N* = 1477) and December 2020 (*N* = 1223), where participants were asked to provide a blood sample and complete a risk factor questionnaire following informed consent.

For this study, the same HCWs were asked to voluntarily participate in a follow-up survey time point one year later in January 2022, following the ‘fourth wave’ of the COVID-19 outbreak in Germany where the Delta variant was reported as dominant and the spread of the Omicron variant had started [17]. This was also following German COVID-19 vaccination recommendations suggesting primary vaccination series (separated by three- or six-weeks) and subsequent booster vaccination (although vaccination for HCWs was not obligatory at the time of the study). Following written informed consent, participants completed a paper questionnaire on sociodemographics, profession and working site, SARS-CoV-2 testing history and more detailed information on the type and timing of COVID-19 vaccination received to-date. A sample of approximately 5 to 6 mL of peripheral venous blood was collected from each participant and stored at + 4 °C until laboratory testing.

The study was prospectively registered with the German Clinical Study Registry (Deutsches Register Klinischer Studien [DRKS]) with DRKS-ID DRKS00027094. The institutional review board of the Berlin Chamber of Physicians (Ärztekammer Berlin, Eth-64/21) provided ethical approval for the study.

### Laboratory procedures

Serological analyses for SARS-CoV-2 were performed using the quantitative SARS-CoV-2 Immunoglobulin G (IgG) enzyme-linked immunosorbent assay (ELISA) with S1 domain substrate (Euroimmun AG, Lübeck, Germany). The assay was applied according to the manufacturer’s recommendations using the following cut-offs: <8 RE/ml (negative), ≥ 8 to < 11 RE/ml borderline, ≥ 11 RE/ml positive. Results in RE/ml were converted to BAU/ml by multiplication with the factor 2.4. Longitudinal samples from those seropositive in May/June and December 2020 were retested using the same method to allow for a valid comparison with the follow-up timepoint in January 2022.

### Statistical analysis

The results of the same HCWs voluntarily participating across the initial and follow-up survey time points were first matched for the analysis. Participants whose blood sample was taken less than fourteen days after their third COVID-19 vaccine dose were excluded (*n* = 3). Missing questionnaire data were not imputed but presented for each variable. Characteristics reported by HCW participants were descriptively summarized with absolute and relative frequencies. Considering the skewed nature of the distribution, SARS-CoV-2 IgG antibody levels were log transformed and presented as geometric mean titres (BAU/mL) with 95% confidence intervals (CIs). Antibody levels were reported according to HCW characteristics including age group, gender, type of profession, time since last COVID-19 vaccine dose, self-reported past SARS-CoV-2 infection, and timing, type and number of COVID-19 vaccine doses received. Kruskal-Wallis test was conducted to find out whether there are statistically significant differences in antibody levels among different groups in HCW characteristics. Two tailed Fisher’s exact test was performed to investigate the relationship between self-reported past SARS-CoV-2 infection rates and selected HCW characteristics. Moreover, logarithmic IgG antibody titres were tested for statistically significant differences in terms of previous laboratory-confirmed SARS-CoV-2 infections, the time since last COVID-19 vaccine dose and the sampling time points using Kruskal-Wallis ANOVA with Dunn’s Test as post-hoc analysis.

Multivariate linear regression was used to further evaluate the association of these characteristics as independent variables and the log transformed IgG antibody levels as the dependent variable among those who had received three COVID-19 vaccine doses.

The log transformed IgG antibody levels were also described by COVID-19 vaccination scheme and the time interval from last COVID-19 vaccination, using Locally Estimated Scatterplot Smoothing (LOESS). The distribution of log transformed IgG antibody levels by COVID-19 vaccination scheme and evidence of previous SARS-CoV-2 infection was shown using boxplots. In order to assess the long-term antibody kinetics, the results from the seropositive HCWs in the previous longitudinal samples in May/June and December 2020 were compared to the most recent follow-up testing in January 2022 in a scatter plot by number of vaccine doses received. Excel version 2019 was used for data entry; R version 4.1.2 (R Foundation for Statistical Computing, Vienna, Austria) and SPSS V27.0 (IBM Deutschland GmbH, Ehningen, Germany) were used for all statistical analyses.

## Results

A total of 1517 HCWs participated in the follow-up serological survey between January 4 and 27, 2022. 73% of participants (*n* = 1112) were women, the median age was 41 years, and approximately half were physicians or nurses (*n* = 729; Table 1). About 15% of participants (*n* = 225) reported a previous laboratory-confirmed SARS-CoV-2 infection by polymerase chain reaction (PCR), of which 5% had more than one infection. Previous SARS-CoV-2 infection was most frequently reported by the youngest participants aged 16 to 29 years (19.1%) and nurses (18.8%) (see Table 2). Almost 80% of HCWs (*n* = 1201) had received three COVID-19 vaccine doses, followed by 13.2% (*n* = 199) with 2 doses, 3.4% (*n* = 51) with one dose and 3.9% (*n* = 58) unvaccinated (Table 1).

The Kruskal-Wallis Test showed that there was no statistically significant difference in antibody levels among gender groups (χ^2^ [3] = 7.594, *p* = 0.052). In all other characteristics of HCW participants, the Kruskal-Wallis test resulted in statistically significant differences, i.e. in terms of age groups (χ^2^ [5] = 27.890, *p* = 0.0001), previous laboratory-confirmed SARS-CoV-2 infection (No/yes, once/yes, multiple times, χ^2^ [2] = 8.489, *p* = 0.0143), type of profession (χ^2^ [4] = 9.004, *p* = 0.0292), Number of vaccine doses received (χ^2^ [8] = 214.076, *p* = 0.0001), combined SARS-CoV-2 vaccination and past infection exposures (χ^2^ [4] = 197.672, *p* = 0.0001), type of specific vaccination received (χ^2^ [8] = 44.654, *p* = 0.0001), type of vaccination scheme received (χ^2^ [3] = 112.851, *p* = 0.0001) and time since last COVID-19 vaccine dose (χ2 [5] = 143.372, *p* = 0.0001).

Fisher’s exact test indicated that there was no significant association between self-reported past SARS-CoV-2 infection rates and age groups (*p* = 0.117), nor between self-reported past SARS-CoV-2 infection rates and gender (*p* = 0.557). However, a significant association was found between infection rates and type of profession (*p* = 0.010).

**Table 1:** SARS-CoV-2 IgG antibody levels (Geometric Mean Titres) according to characteristics of HCW participants following COVID-19 vaccination (*N* = 1517)

Characteristic	*n* (%)	Geometric Mean Titres¥ (95% CI)
Age groups in years		
16–29*	267 (17.6%)	1470 (1286–1679)
30–39	425 (28.0%)	1112 (986–1256)
40–49	360 (23.7%)	1319 (1151–1513)
50–59	338 (22.3%)	1423 (1275–1589)
60+	99 (6.5%)	1756 (1477–2087)
Unknown	28 (1.8%)	833 (466–1487)
Gender		
Female	1112 (73.3%)	1344 (1251–1445)
Male	397 (26.2%)	1233 (1105–1377)
Non-Binary	4 (0.3%)	1769 (1131–2766)
Unknown	4 (0.3%)	2167 (658–7134)
Previous laboratory-confirmed SARS-CoV-2 infection		
Yes, once	214 (14.1%)	1487 (1265–1747)
Yes, > 1 reinfection	11 (0.7%)	1776 (970–3251)
Time since previous laboratory confirmed SARS-CoV-2 infection		
≤ 3 months	58 (3.8%)	1507 (1103–2059)
> 3 months	153 (10.1%)	1524 (1270–1829)
unknown	14 (0.9%)	1227 (468–3220)
No	1284 (84.6%)	1285 (1203–1372)
Unknown	8 (0.5%)	1845 (937–3630)
Type of profession		
Nurse	467 (30.8%)	1309 (1167–1468)
Physician	262 (17.3%)	1278 (1144–1428)
Other allied health professionals	402 (26.5%)	1382 (1229–1555)
Administration/other facility management	364 (24.0%)	1272 (1117–1449)
Unknown	22 (1.5%)	1577 (879–2829)
Number of vaccine doses received		
Three times vaccinated (*plus* evidence of past SARS-CoV-2 infection)	79 (5.2%)	2204 (1919–2531)
Three times vaccinated (no evidence of past SARS-CoV-2 infection)	1122 (74.0%)	1650 (1585–1717)
Two times vaccinated (*plus* evidence of past SARS-CoV-2 infection)	98 (6.5%)	1755 (1433–2150)
Two times vaccinated (no evidence of past SARS-CoV-2 infection)	101 (6.7%)	335 (258–434)
One time vaccinated (*plus* evidence of past SARS-CoV-2 infection)**	32 (2.1%)	1059 (713–1573)
One time vaccinated (no evidence of past SARS-CoV-2 infection)***	19 (1.3%)	394 (132–1176)
Not vaccinated (*plus* evidence of past SARS-CoV-2 infection)	16 (1.1%)	171 (61–475)
Not vaccinated (no evidence of past SARS-CoV-2 infection)	42 (2.8%)	69 (26–184)
Unknown	8 (0.5%)	1845 (937–3630)
Combined SARS-CoV-2 vaccination and past infection exposures		
None	42 (2.8%)	69 (26–184)
One exposure	34 (2.3%)	272 (128–578)
Two exposures	133 (8.8%)	435 (345–549)
Three exposures	1217 (80.6%)	1655 (1590–1722)
Four exposures	79 (5.2%)	2260 (1962–2602)
Five exposures	4 (0.3%)	2043 (1366–3056)
Type of specific vaccination received (among those three times vaccinated)	*N* = 1201	
2 doses BNT162b2 (3 weeks apart) + BNT162b2 booster	436 (36.3%)	1629 (1530–1734)
2 doses BNT162b2 (6 weeks apart) + BNT162b2 booster	130 (10.8%)	2008 (1761–2291)
1 dose ChAdOx1-S, 1 dose BNT162b2 + BNT162b2 booster	378 (31.5%)	1576 (1478–1681)
2 doses ChAdOx1-S + BNT162b2 booster	159 (13.2%)	1699 (1505–1918)
1 dose ChAdOx1-S, 1 dose BNT162b2 + mRNA-1273 booster	23 (1.9%)	2154 (1788–2595)
2 doses mRNA-1273 + BNT162b2 booster	11 (0.9%)	1733 (1305–2300)
2 doses BNT162b2 + mRNA-1273 booster	11 (0.9%)	3343 (2311–4837)
2 doses ChAdOx1-S + mRNA-1273 booster	11 (0.9%)	3502 (2338–5246)
Unknown/other vaccine scheme	42 (3.5%)	1472 (1244–1741)
Type of vaccination scheme received (among those three times vaccinated)		
Homologous COVID-19 vaccine scheme	566 (47.1%)	1710 (1615–1810)
Heterologous COVID-19 vaccine scheme with mRNA-1273 booster	45 (3.7%)	2657 (2267–3114)
Heterologous COVID-19 vaccine scheme with another booster	548 (45.6%)	1614 (1525–1709)
Time since last COVID-19 vaccine dose (among those three times vaccinated)	*N* = 1201	
< 30 days	78 (6.5%)	2151 (1698–2724)
30–49 days	586 (48.8%)	1877 (1786–1971)
50–69 days	299 (24.9%)	1627 (1510–1754)
70–89 days	170 (14.2%)	1324 (1200–1461)
≥ 90 days	37 (3.1%)	1156 (876–1524)
Unknown	31 (2.6%)	1249 (857–1821)

Abbreviations: CI: Confidence Interval; IgG: Immunoglobulin G

¥ SARS-CoV-2 Immunoglobulin G (IgG) antibody levels expressed as geometric mean titres (BAU/mL) with 95% confidence intervals (CIs)

*Includes those aged 16–18 years worked as interns or on their practicums in the hospital system

**N = 4 received Ad26.COV2.S and *N* = 28 other vaccines; The four participants who received one dose of Ad26.COV2.S had IgG geometric means (+ 95% CI) of 766 (65-8976)

***N = 4 received Ad26.COV2.S and *N* = 15 other vaccines; The four participants who received one dose of Ad26.COV2.S had IgG geometric means (+ 95% CI) of 12 (1-174)

**Table 2:** Proportion of HCWs who self-reported past SARS-CoV-2 infection by selected characteristics (*N* = 1517)

Characteristic	Self-reported past SARS-CoV-2 infection	
	*n*	% (95% CI)
Age groups in years		
16–29 (*N* = 267)	51	19.1 (14.4–23.8)
30–39 (*N* = 425)	61	14.4 (11.0-17.7)
40–49 (*N* = 360)	51	14.2 (10.6–17.8)
50–59 (*N* = 338)	40	11.8 (8.4–15.3)
60+ (*N* = 99)	15	15.2 (8.1–22.2)
Unknown (*N* = 28)	7	25.0 (9.0–41.0)
Gender		
Female (*N* = 1112)	164	14.7 (12.7–16.8)
Male (*N* = 397)	59	14.9 (11.4–18.4)
Non-Binary (*N* = 4)	1	25.0 (-17.4-67.4)
Unknown (*N* = 4)	1	25.0 (-17.4-67.4)
Type of profession		
Nurse (*N* = 467)	88	18.8 (15.3–22.4)
Physician (*N* = 262)	43	16.4 (11.9–20.9)
Other allied health professionals (*N* = 402)	44	10.9 (7.9–14.0)
Administration/other facility management (*N* = 364)	46	12.6 (9.2–16.1)
Unknown (*N* = 22)	4	18.2 (2.1–34.3)

Abbreviations: HCW: Health Care Worker, n: number of Participants, CI: Confidence Interval

Among those who had received three vaccine doses, 92% received a vaccination scheme including the BNT162b2 (Comirnaty, BioNTech/Pfizer) and/or ChAdOx1-S (Vaxzevria, Oxford/AstraZeneca) vaccines. Other vaccination schemes also included the mRNA-1273 (Spikevax, Moderna) or Ad26.COV2-S (Janssen/Johnson&Johnson) vaccines (Table 1). The median number of days between the second and third vaccine dose ranged from 182 to 265 days and the median number of days between the third vaccine dose and survey time of testing was 37 to 65 days. In order to investigate whether different time intervals between booster vaccination and blood sampling in age groups, gender or type of profession could have an influence on the level of GMTs of the respective groups, the GMTs were grouped according to time intervals < 1 month, 1–2 months, >2months. This clearly shows that nurses, physicians and older patients were vaccinations given longer ago and that the 3 doses of BNT162b2 (primary series separated by 6 weeks) vaccine scheme was administered earlier compared to other vaccine schemes (Appendix Table 1  and Appendix Table 2).

### Estimates of SARS-CoV-2 IgG antibody levels

In January 2022, 1482 (97.7%) HCWs tested SARS-CoV-2 seropositive, whereas 29 (1.9%) and 6 (0.4%) were negative and borderline, respectively. This represented a large increase from the previous year, where 4.6% tested seropositive prior to the first vaccinations offered at the hospital in December 2020. Among the 35 HCWs with negative or borderline results, 29 (82.9%) were unvaccinated or had only been vaccinated one time, including 4/29 (13.8%) who had reported evidence of past SARS-CoV-2 infection but were still testing IgG negative or borderline. Only one out of 1201 HCWs who had received three vaccine doses had a borderline IgG antibody result (Appendix Table 3).

**Fig. 1:**
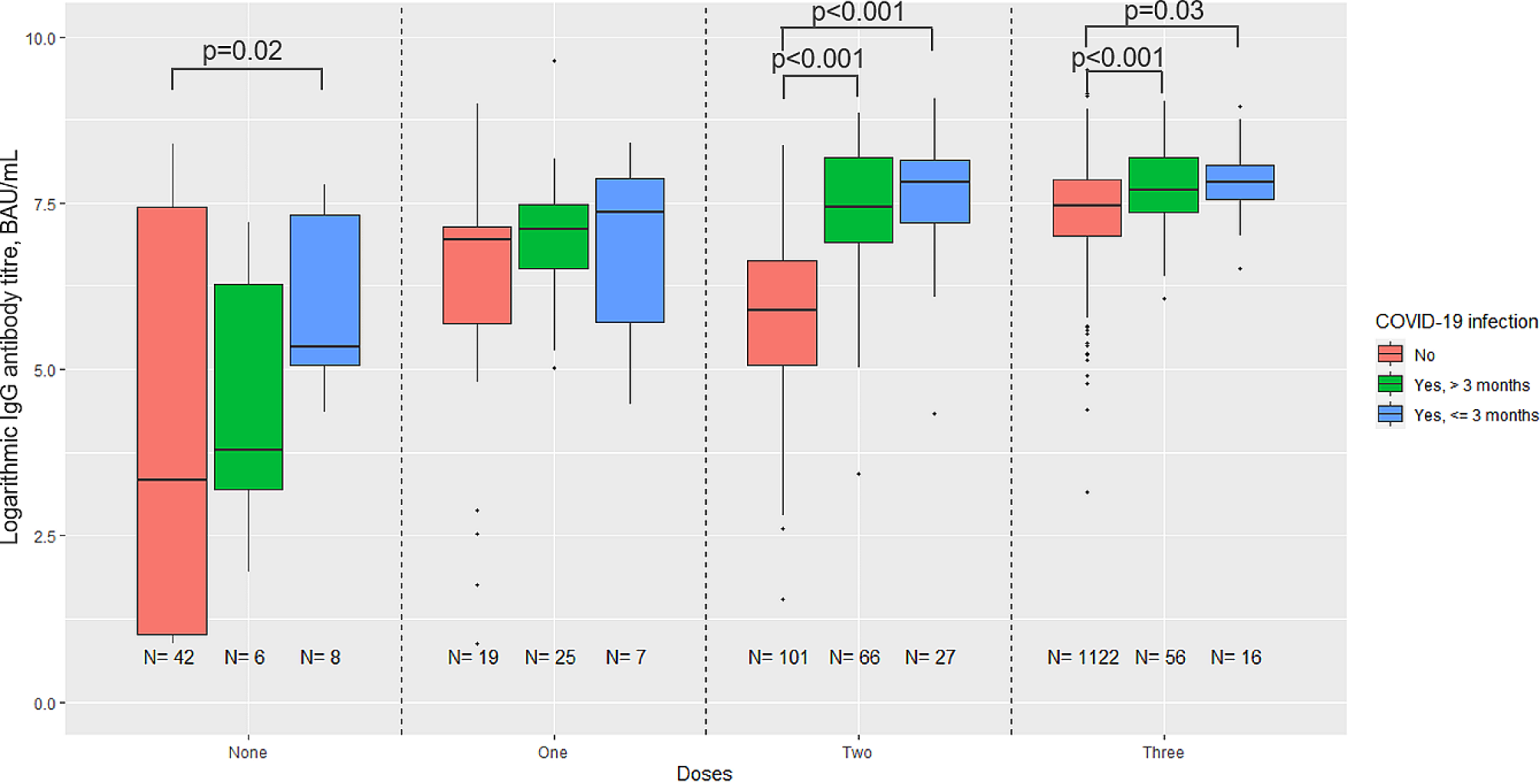
SARS-CoV-2 IgG antibody levels (logarithmic titres, BAU/ml) by COVID-19 vaccination status and evidence of previous laboratory-confirmed SARS-CoV-2 infection (*N* = 1495) Boxplots showing medians, interquartile ranges and 95% confidence intervals of logarithmic IgG antibody titres by COVID-19 vaccinations status and evidence of SARS-CoV-2 infection. *P*-values are only shown for statistically significant differences (i.e., *p*-value < 0.05) between groups estimated by Kruskal-Wallis ANOVA using Dunn’s test for pairwise multiple comparisons

The overall IgG geometric mean titre (GMT) of the entire cohort was 1317 BAU/mL (95% CI: 1240–1399). This ranged from 2204 (95% CI: 1919–2531) among participants with hybrid immunity from three vaccine doses and an evidence of past SARS-CoV-2 infection to 69 (95% CI: 26–184) among those without vaccination and no evidence of previous SARS-CoV-2 infection (Table 1; Fig. 1). Among HCWs without evidence of past SARS-CoV-2 infection, those vaccinated three times against COVID-19 had a GMT (1650, 95% CI: 1585–1717) approximately 5 times higher than HCWs vaccinated only with the 2-dose primary vaccination series (335, 95% CI: 258–434). Although HCWs with evidence of previous SARS-CoV-2 infection but unvaccinated had a substantially lower GMT (171, 95% CI: 61–475), those who had only two vaccine doses plus evidence of previous infection had a comparable GMT (1755, 95% CI: 1433–2150) to those vaccinated three times and no evidence of previous infection (1650, 95% CI: 1585–1717). Among HCWs who received two or three vaccine doses, the GMTs were significantly higher at the significance level of alpha = 0.05 among participants with evidence of infection than among participants with no evidence of infection (Fig. 1).

The grouping of GMTs according to SARS-CoV-2 exposure, in which all infections and vaccine doses of a participant are added together, shows a similar picture. GMTs increase constantly with rising SARS-CoV-2 exposure from 69 (95% CI: 26–184, no exposure) to 2260 (95% CI: 1962–2602, four exposures), whereby the highest exposure (i.e., three vaccine doses and two SARS-CoV-2 infections) cannot be regarded as representative due to the small number of participants in this group (see Table 1).

The timing of the last SARS-CoV-2 infection did not seem to have a major impact on the IgG antibody levels (see Appendix Table 4).

**Fig. 2:**
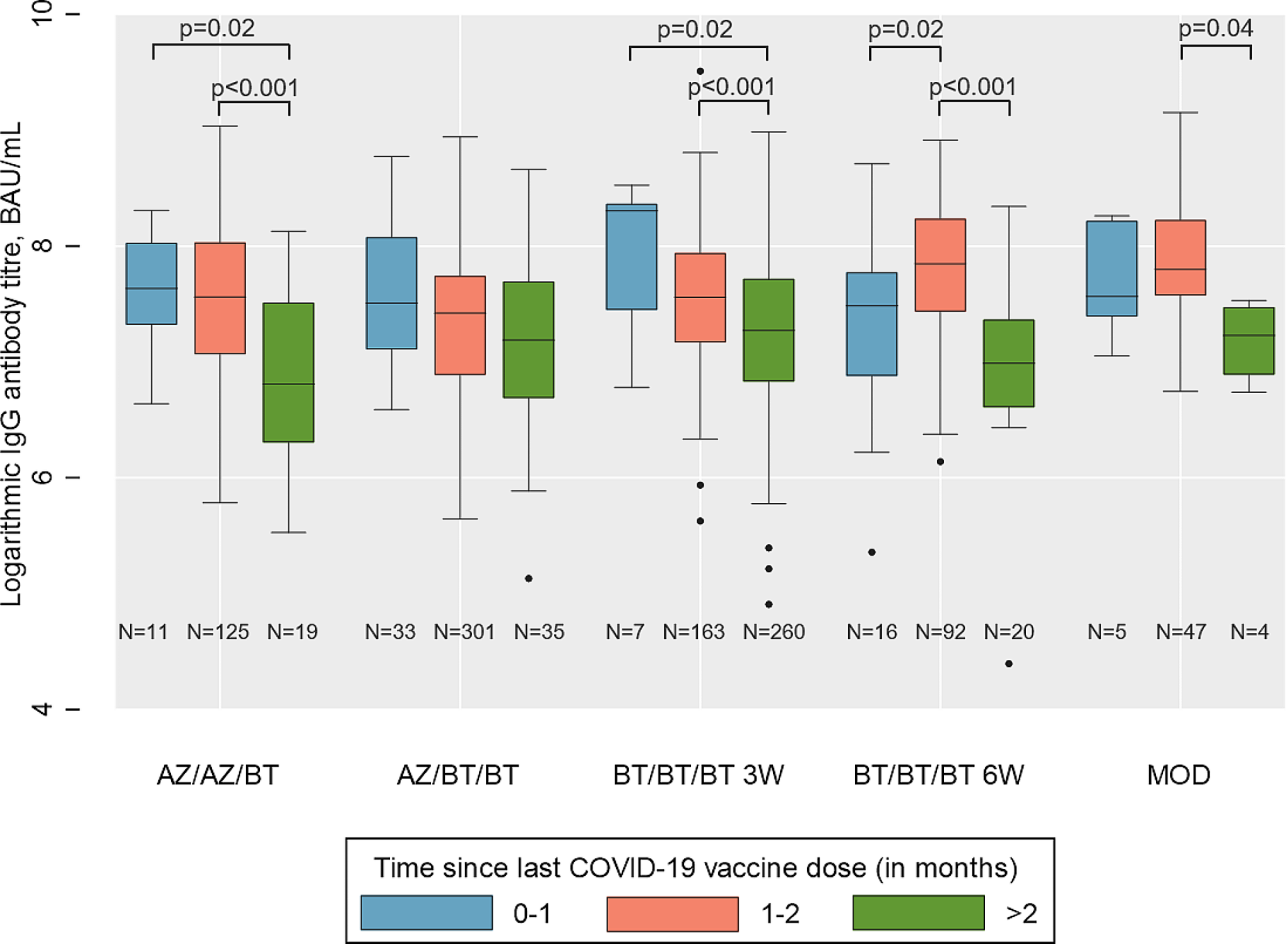
SARS-CoV-2 IgG antibody levels (logarithmic titres, BAU/mL) by type of COVID-19 vaccination among those vaccinated three times (*N* = 1138) Boxplots showing medians, interquartile ranges and 95% confidence intervals of logarithmic IgG antibody titres by time since last COVID-19 vaccine dose and type of COVID-19 vaccination. *P*-values are only shown for statistically significant differences (i.e., *p*-value < 0.05) between groups estimated by Kruskal-Wallis ANOVA using Dunn’s test for pairwise multiple comparisons. Abbreviations: IgG: Immunoglobulin G, N: Number of Participants, Vaccination schemes: AZ/AZ/BT = 2 doses ChAdOx1-S + BNT162b2 booster (*N* = 155), AZ/BT/BT = 1 dose ChAdOx1-S, 1 dose BNT162b2 + BNT162b2 booster (*N* = 369), BT/BT/BT 3 W = 2 doses BNT162b2 (3 weeks apart) + BNT162b2 booster (*N* = 430), BT/BT/BT 6 W = 2 doses BNT162b2 (3 weeks apart) + BNT162b2 booster (*N* = 128), MOD = Vaccine combinations with at least one dose mRNA-1271 (*N* = 56)

Although fewer HCWs received heterologous COVID-19 vaccination combinations including mRNA-1273, those who had received mRNA-1273 as the third booster dose had the highest GMTs, an average of 2657 (95% CI: 2267–3114) compared to an average of 1710 (95% CI: 1615–1810) among homologous vaccination schemes and an average of 1614 (95% CI: 1525–1709) among other heterologous vaccination schemes without mRNA-1273 as the third booster dose (Table 1). The highest GMT was 3502 (95% CI: 2338–5246) among those who received two doses of ChAdOx1-S followed by a third dose of mRNA-1273. Among HCWs who had received 3 doses of BNT162b2, those who had received the 2 dose-primary series separated by 6 weeks had a higher GMT (2008, 95% CI: 1761–2291) compared to those where it was separated by 3 weeks (1629, 95% CI: 1530–1734). However, the median number of days between the booster and the primary series separated by 3 weeks (265) was greater than that of the primary series separated by 6 weeks (183; Appendix Table 2). The decline in IgG titres since the last COVID-19 vaccine dose for each vaccination schedule can be seen in Fig. 2 and Appendix Fig. 1.

### Factors associated with log-transformed SARS-CoV-2 IgG antibody levels

In the multivariate model, HCWs aged 16–29 had significantly higher IgG antibody levels compared to those with 30–39 years (*p* < 0.01), 40–49 years (*p* < 0.05), and 50–59 years (*p* < 0.05; Table 3).

**Table 3:** Multivariate linear regression model assessing factors associated with IgG antibody levels (logarithmic titres, BAU/mL) among those vaccinated three times (*N* = 1124^*^)

Covariates	*n* (%)	Regression Coefficient	95% CI	*p*-value**
Age in years				
16–29	182	Reference	--	--
30–39	308	**-0.170**	**-0.287 – -0.052**	**< 0.01**
40–49	275	**-0.126**	**-0.247 – -0.004**	**< 0.05**
50–59	275	**-0.146**	**-0.272 – -0.020**	**< 0.05**
60+	84	-0.174	-0.351–0.003	0.054
Gender				
Male	309	Reference	--	**--**
Female	815	0.083	-0.006–0.171	0.067
Type of profession				
Administration/Other facility management	263	Reference	--	--
Nurse	329	**-0.134**	**-0.252–0.015**	**< 0.05**
Physician	219	**-0.254**	**-0.379 – -0.128**	**< 0.001**
Other allied health professionals	313	-0.100	-0.210–0.010	0.076
Positive PCR test result for COVID-19				
No	955	Reference		
Yes, <=3 months	62	**0.437**	**0.101–0.773**	**< 0.01**
Yes, > 3 months	107	**0.308**	**0.132–0.484**	**< 0.001**
Time since last COVID-19 vaccine dose				
≤ 50 days	635	Reference		
> 50 days	489	**-0.243**	**-0.334 - -0.152**	**< 0.001**
Vaccination				
2 doses BNT162b2 (3 weeks apart) + BNT162b2 booster	428	Reference	--	--
2 doses BNT162b2 (6 weeks apart) + BNT162b2 booster	124	0.035	-0.102–0.173	0.615
1 dose ChAdOx1-S, 1 dose BNT162b2 + BNT162b2 booster	366	**-0.239**	**-0.350 – -0.129**	**< 0.001**
2 doses ChAdOx1-S + BNT162b2 booster	155	**-0.150**	**-0.280 – -0.024**	**< 0.05**
Vaccine combinations with at least one dose mRNA-1273***	51	**0.235**	**0.048–0.422**	**< 0.05**

Abbreviation: CI: Confidence Interval

*Due to missing values in each of the covariates and exclusion of minor categories (i.e. non-binary gender, rare combinations of vaccines received) the number of participants reduces from 1201 participants with three vaccines received to 1124

**Bold represents *p* < 0.05

***These include: 1 dose ChAdOx1-S, 1 dose BNT162b2 + mRNA-1273 booster, 2 doses mRNA-1273 + BNT162b2 booster, 2 doses BNT162b2 + mRNA-1273 booster, 2 doses ChAdOx1-S + mRNA-1273 booster

Moreover, physicians and nurses were found to have − 0.254 (95% CI: -0.379- -0.128; *p* < 0.001) and − 0.134 (95% CI: -0.252- -0.015, *p* < 0.05) lower IgG antibody levels, respectively, compared to administration or other facility management staff. HCWs who reported a previous COVD-19 infection ≤ 3 months and > 3 months before the time of testing had 0.437 (95% CI: 0.101–0.773; *p* < 0.01) and 0.308 (95% CI: 0.132–0.484; *p* < 0.001) higher IgG antibody levels, respectively, compared to those with no evidence of past infection. Those who had received the third COVID-19 vaccine dose more than 50 days before the time of testing had − 0.243 (95% CI: -0.334- -0.152; *p* < 0.001) lower, IgG antibody levels compared to those who received it 50 days before or less.

Similar to the results of the univariate GMT estimates, HCWs who had received heterologous COVID-19 vaccination combinations including mRNA-1273 as the booster had significantly higher IgG antibody levels compared to those received 3 doses of BNT162b2 (*p* < 0.05). After accounting for the other variables in the model, there was no longer a significant difference in IgG antibody levels between those receiving the 2 dose-primary series of BNT162b2 separated by 6 weeks compared to 3 weeks (*p* = 0.615). However, the heterologous COVID-19 vaccination combinations of ChAdOx1-S and BNT162b2 had significantly lower IgG antibody levels compared to the 3 dose series of BNT162b2 with the primary series separated by 3 weeks (*p* < 0.05).

### Longitudinal results of IgG antibody levels

**Fig. 3:**
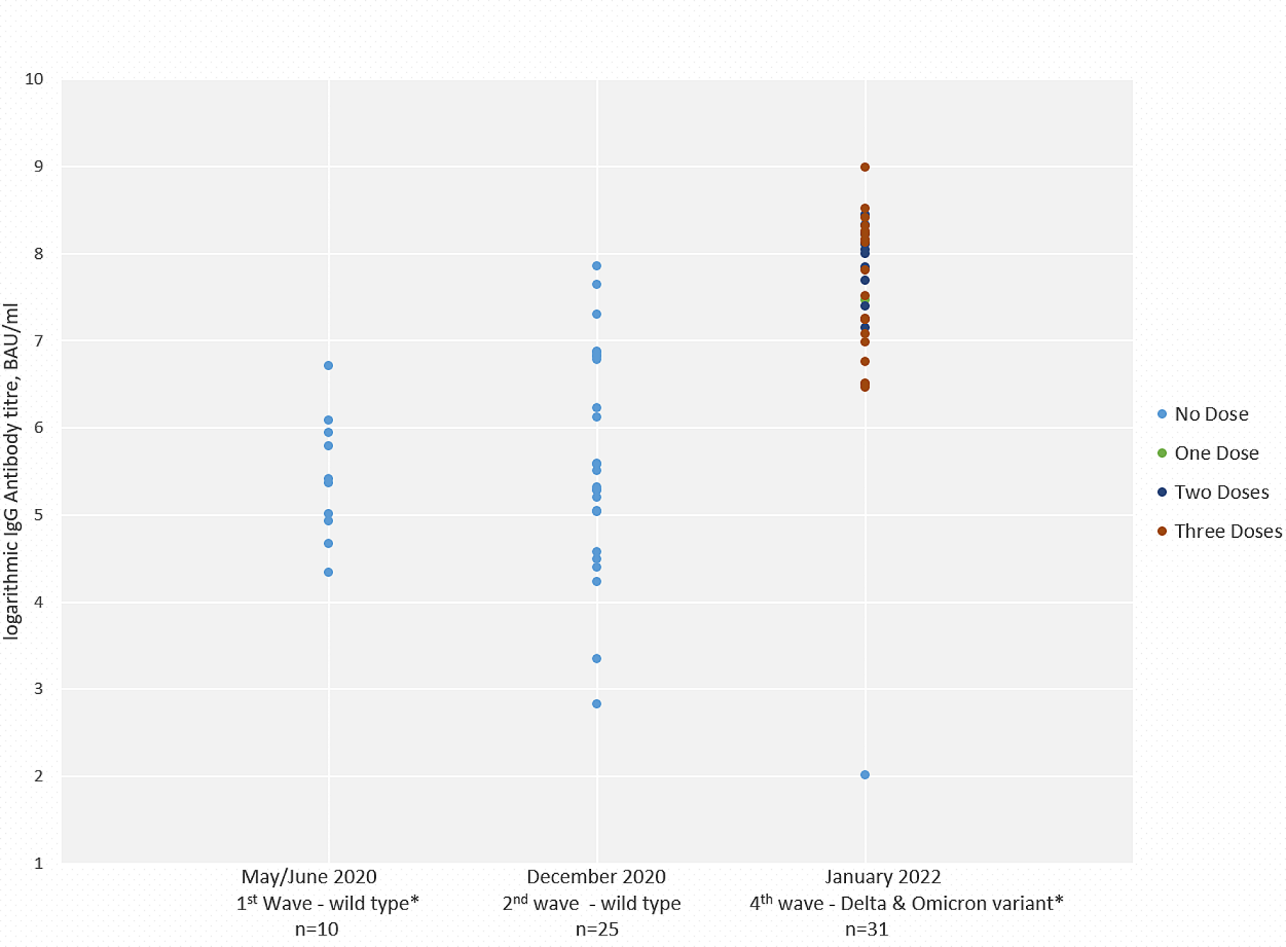
Longitudinal SARS-CoV-2 IgG antibody levels (logarithmic titres, BAU/mL) among those initially seropositive in the May/June 2020 or December 2020 timepoints by vaccination status (*N* = 31) * In Germany: (1) May/June 2020 was following the first wave where the wild-type SARS-CoV-2 was reported as dominant; (2) December 2020 was in the second wave where the wild-type SARS-CoV-2 was reported as dominant; (3) the third wave occurred in Spring 2021 where the wild-type SARS-CoV-2 and Alpha variant were reported as dominant; and (4) January 2022 was following the fourth wave where the Delta variant was reported as dominant and where the spread of the Omicron variant was reported before the fifth wave [17], Abbreviations: IgG - Immunoglobulin G, n – number of Participants

Thirty-one HCWs with laboratory-confirmed SARS-CoV-2 infections who tested positive starting in May 2020 (pre-COVID-19 vaccination) or in December 2020 were followed up in the present survey in January 2022 (post-COVID-19 vaccination). Among these HCWs, the GMT of IgG antibody levels was 226 BAU/mL (95% CI: 135–378) in May/June 2020, 281 BAU/mL (95% CI: 165–479) in December 2020, and 2226 BAU/mL (95% CI: 1717–2885) in January 2022 (Fig. 3). This reflected an 8- to 10-fold increase in January 2022 compared with the two previous time points. Pairwise comparisons using Dunn’s test showed that logarithmic IgG antibody titres in January 2022 were observed to be significantly different from those in May 2020 (z=-4.998, *p* < 0.0001) and December 2020 (z=-4.350, *p* < 0.0001). The difference in logarithmic IgG antibody titres between May 2020 and December 2020 were not statistically significant (z=-0.638, *p* = 1.0000). Only one HCW who was initially seropositive in May 2020 did not receive any vaccination against SARS-CoV-2 and turned IgG seronegative at the 2022 time point.

## Discussion

We report the results of a follow-up serological survey among HCWs at a large tertiary COVID-19 referral hospital following a longitudinal investigation from 2020 to 2022. Although the dynamics of the COVID-19 pandemic have continued to evolve, we believe that the findings still contribute to the evidence on the magnitude of immunological protection induced from varying COVID-19 vaccination schemes and exposure to SARS-CoV-2 infection. As expected, a large increase in seropositivity was seen from 1.2% in May/June 2020 (following the first COVID-19 wave in Germany) and 4.6% in December 2020 (during the second wave) to 97.7% in January 2022 (following the fourth wave including the Omicron variant), where 80% of participating HCWs had then received three vaccine doses and 15% had reported a previous SARS-CoV-2 infection at the time of the survey. Heterologous COVID-19 vaccination combinations including a mRNA-1273 booster were significantly associated with the highest IgG antibody levels, and vaccination schemes with three doses of BNT162b2 also resulted in higher IgG antibody levels compared to other heterologous three dose schemes with BNT162b2 and ChAdOx1-S. Those with hybrid immunity from COVID-19 vaccination and previous SARS-CoV-2 infection had the highest immunological protection. This included an 8- to 10-fold increase in IgG antibody levels among HCWs who reported a previous SARS-CoV-2 infection in May 2020 to January 2022 after COVID-19 vaccination, emphasising the importance of vaccination for previously infected individuals.

The estimated SARS-CoV-2 seropositivity of 97.7% among HCWs in our survey was slightly higher than the findings of a national survey in the general population among adults in Germany which found an estimate of 92% at the beginning of 2022 as well as a European multi-country study of HCWs which found a seroprevalence of more than 90% [18, 19]. In this multi-country study by the European Centre for Disease Prevention and Control (ECDC) which was conducted in 16 hospital sites across Croatia, Estonia, Greece, Ireland, Italy, Latvia, Poland, Portugal, and Spain, 64% of participating HCWs had been vaccinated with any booster dose by July 2022 [18], an estimate lower than in our study where 80% had been vaccinated by January 2022. Likewise, when compared with the general population in Germany where approximately 44% of adults were estimated to have been vaccinated with a booster at the beginning of 2022, the vaccination rate among HCWs in our survey was also substantially higher [19]. Within the health care facility setting, other surveys in Germany showed that HCWs who had greater patient contact and COVID-19 exposure risk were more likely to report agreement with COVID-19 booster vaccination [20].

Our study showed that all three heterologous vaccine combinations with the mRNA-1273 booster resulted in significantly higher IgG antibody levels of participating HCWs, although vaccination schemes with three doses of BNT162b2 also produced higher IgG antibody levels compared to other heterologous schemes with BNT162b2 and ChAdOx1-S. A systematic review by Mojadadi et al., including three randomized controlled trials (RCTs) comparing heterologous and homologous vaccination regimes, found the highest antibody response for the 3-dose homologous vaccination regimen of mRNA-1273, followed by the 2 dose-primary series of BNT162b2 combined with the mRNA-1273 booster [21]. An earlier review of immunogenicity studies by Cheng et al. reported that a mRNA-1273 booster after primary doses of viral vector or inactivated vaccines increased the antibody response compared to other vaccination regimens [22]. Our study also showed no significant differences between HCWs who received a 2-dose primary series of BNT162b2 separated by 3 or 6 weeks, when followed by a third booster of BNT162b2. These results further demonstrate the value of mRNA booster vaccines for ongoing COVID-19 vaccination strategies.

These findings are also important when considering breakthrough infections of recent Omicron variants which are occurring at higher IgG antibody levels compared to previous variants. Regenhardt et al. reported that HCWs in a hospital in Germany experienced Omicron breakthrough infections at a median IgG antibody level of 1235, whereas Delta breakthrough infections occurred at a median IgG antibody level of 138 [8]. Moreover, Mohlendick et al. estimated that individuals with an IgG antibody level of 2816 BAU/ml or less had a 2-fold increased risk for a breakthrough infection compared to study participants exceeding this cut-off [9]. Thus, a mRNA-1273 booster vaccine appears to be an important measure in preventing infections with the Omicron variants, particularly among at-risk populations.

As reported in other studies, our analysis found that hybrid immunity from COVID-19 vaccination and prior laboratory confirmed SARS-CoV-2 infection was found to be associated with higher IgG antibody levels [23–29]. However, HCWs with evidence of previous SARS-CoV-2 infection but unvaccinated had substantially lower IgG antibody levels and the longitudinal analysis of HCWs with prior infection measured in 2020 showed an 8- to 10-fold increase in IgG antibody levels after administration of the booster vaccine in 2022. As the COVID-19 pandemic continues to evolve and population immunity from previous infection continues to spread, these findings highlight the need to consider booster vaccination, particularly among at-risk populations.

While HCWs with higher occupational risk had significantly higher IgG antibody levels in the pre-vaccination period as demonstrated in our earlier study [16] as well as in other studies [30], physicians and nurses had significantly lower IgG antibody levels after booster vaccination compared to administrative or other facility management staff in this current analysis. Although we controlled for the time of last COVID-19 vaccine dose ≤ or > 50 days in the multivariate analysis, they may be due to residual differences in the timing of booster vaccinations for physicians and nurses who received their booster vaccinations earlier compared to other staff without direct patient contact. The earlier administration, thus, led to a larger interval between last vaccine dose and blood sampling. Those who had received their last COVID-19 vaccine dose > 50 days in the past had significantly lower IgG antibody levels in the multivariate model. These findings again emphasize the need to carefully consider timing of COVID-19 booster strategies for populations such as HCWs with direct patient contact.

We also found a significant association between increasing age and SARS-CoV-2 IgG antibody levels but no differences across gender, adding to some mixed evidence in this area. Increasing age has been shown to be associated with increased IgG antibody levels in various individual studies [31], although a subgroup analysis of a meta-analysis [22] and a longitudinal study of HCWs in Romania did not find significant differences in antibody concentrations with respect to age [32]. Our comparable findings in men and woman were also found in the national SOEP study in Germany conducted by Robert Koch Institut [19]. In contrast, various studies reported an association between male gender and lower SARS-CoV-2 serological levels [33–39]. Possible explanations for these mixed findings may be due to the application of different serological assays and varying other uncontrolled background characteristics across studies.

Our study has the following limitations that should be considered: Although our study’s cohort reflects the full population of HCWs at the study hospital well in regards to demographics, type of profession, and working location, the voluntary participation in the study may still have led to sample bias. Secondly, questionnaire responses concerning past vaccination or other exposures could have been affected by recall and social desirability biases, although pseudonymized data collection hopefully minimized this risk. Thirdly, we did not determine neutralizing antibody titres for all HCWs in this analysis, which may have shown varying immune responses. Furthermore, a fraction of patients may have unknowingly had a SARS-CoV-2 infection that was not captured by the self-reported questionnaire or IgG ELISA results. However, routine SARS-COV-2 testing was offered at the facility so this could have reduced such bias. Lastly, our longitudinal cohort was small with 31 HCWs who were initially seropositive in 2020 due to the lower seropositivity rate early in the pandemic, affecting the reliability of these conclusions.

## Conclusions

In summary, this analysis of a follow-up study among health care workers at a large tertiary hospital in Germany adds to the body of evidence on immunological protection induced from different COVID-19 vaccination schemes and previous infection in different settings. It emphasises the importance of ongoing COVID-19 booster vaccination strategies, including careful consideration of the most effective type of vaccination and timing of administration for at-risk populations, when feasible. As studies have shown, breakthrough infections of variants such as Omicron can occur at considerably high antibody levels, highlighting both the continued importance of protective measures against SARS-CoV-2 infections as well as longitudinal testing of antibody levels to assess the risk of breakthrough infections and evolving population scenarios of hybrid immunity.

### Electronic supplementary material

Below is the link to the electronic supplementary material.

Supplementary Material 1

## Data Availability

The datasets used and/or analysed during the current study are available from the corresponding author on reasonable request.

## Abbreviations

CI: confidence intervals
COVID-19: coronavirus disease 2019
DRKS: Deutsches Register Klinischer Studien [engl.: *German Clinical Study Registry*]
ECDC: European Centre for Disease Prevention and Control
EEA: European Economic Area
ELISA: enzyme-linked immunosorbent assay
EU: European Union
GMT: geometric mean titre
HCW: health care worker
IgG: Immunoglobulin G
LOESS: Locally Estimated Scatterplot Smoothing
n –: Number of Subjects
PCR: polymerase-chain reaction
RCT: randomized controlled trial
SARS-CoV-2: severe acute respiratory syndrome coronavirus-2
ukb: Unfallkrankenhaus Berlin
WHO: World Health Organization
