# Supplementary material for: Follow-up SARS-CoV-2 serological study of a health care worker cohort following COVID-19 booster vaccination
Source: BMC Infect Dis. 2024 Apr 24;24:436. doi: 10.1186/s12879-024-09338-5 (PMC11040945; doi:10.1186/s12879-024-09338-5)
Supplement: Supplementary file 1 — Supplementary Material 1 [file 12879_2024_9338_MOESM1_ESM.docx]

**Appendix Table 1. SARS-CoV-2 IgG antibody levels according to characteristics of HCW participants, grouped by time since COVID-19 booster vaccination (N=1185)**

|  | **Time since COVID-19 booster vaccination** | | | | | |
| --- | --- | --- | --- | --- | --- | --- |
|  | **<1 month** | | **1-2 months** | | **>2 months** | |
| **Characteristic** | **n (%)** | **GMT¥** | **n (%)** | **GMT¥** | **n (%)** | **GMT¥** |
| Age groups in years |  |  |  |  |  |  |
| 16-29 | 18 (23%) | 2417 | 146 (19%) | 1882 | 28 (8%) | 2057 |
| 30-39 | 23 (29%) | 1654 | 215 (28%) | 1691 | 83 (24%) | 1295 |
| 40-49 | 16 (20%) | 1654 | 183 (24%) | 1915 | 88 (25%) | 1348 |
| 50-59 | 16 (20%) | 2343 | 160 (21%) | 1789 | 105 (30%) | 1364 |
| 60+ | 6 (8%) | 1558 | 45 (6%) | 2245 | 36 (10%) | 1338 |
| Unknown | 1 (1%) | 1414 | 8 (1%) | 1784 | 8 (2%) | 1436 |
| Gender |  |  |  |  |  |  |
| Female | 61 (76%) | 1898 | 573 (76%) | 1917 | 227 (65%) | 1398 |
| Male | 19 (24%) | 1987 | 181 (24%) | 1578 | 119 (34%) | 1365 |
| Non-Binary | 0 |  | 1 (0.1%) | 2696 | 2 (1%) | 1542 |
| Unknown | 0 |  | 2 (0.3%) | 2397 | 0 |  |
| Type of profession |  |  |  |  |  |  |
| Nurse | 18 (23%) | 2098 | 176 (23%) | 1318 | 151 (43%) | 1421 |
| Physician | 8 (10%) | 1437 | 128 (17%) | 501 | 88 (25%) | 532 |
| Other allied health professionals | 22 (28%) | 1913 | 238 (31%) | 1886 | 67 (19%) | 1411 |
| Administration/other facility management | 31 (39%) | 1975 | 205 (27%) | 1918 | 37 (11%) | 1377 |
| Unknown | 1 (1%) | 1714 | 10 (1%) | 2051 | 5 (1%) | 1636 |
| Type of vaccination received* |  |  |  |  |  |  |
| 2 doses BNT162b2 (3 weeks apart) + BNT162b2 booster | 7 (10%) | 2953 | 163 (24%) | 1912 | 261 (78%) | 1466 |
| 2 doses BNT162b2 (6 weeks apart) + BNT162b2 booster | 16 (24%) | 1534 | 92 (14%) | 2480 | 19 (6%) | 1031 |
| 1 dose ChAdOx1-S, 1 dose BNT162b2 + BNT162b2 booster | 34 (50%) | 1886 | 301 (44%) | 1559 | 36 (11%) | 1349 |
| 2 doses ChAdOx1-S + BNT162b2 booster | 11 (16%) | 2032 | 125 (18%) | 1884 | 19 (6%) | 917 |

Abbreviations: IgG: Immunoglobulin G, HCW: Health Care Worker, n: number of Participants, GMT: Geometric Mean Titres,

¥ SARS-CoV-2 Immunoglobulin G (IgG) antibody levels expressed as geometric mean titres (BAU/mL)

*Only the four main vaccination schemes received are displayed (N=1084)

**Appendix Table 2. Days between doses when three vaccines received (N=1201)**

|  |  | **Median days between (IQR)** | | |
| --- | --- | --- | --- | --- |
| **Type of vaccination received** | **n** | **Dose 1 & Dose 2** | **Dose 2 &**  **Dose 3** | **Dose 3 &**  **Blood sample** |
| 2 doses BNT162b2 (3 weeks apart) + BNT162b2 booster | 436 | 21 (21-22) | 265 (255-278) | 65 (53-78) |
| 2 doses BNT162b2 (6 weeks apart) + BNT162b2 booster | 130 | 42 (35-42) | 183 (171-208) | 41 (36-50) |
| 1 dose ChAdOx1-S, 1 dose BNT162b2 + BNT162b2 booster | 378 | 84 (82-86) | 196 (189-202) | 41 (35-48) |
| 2 doses ChAdOx1-S + BNT162b2 booster | 159 | 82 (80-84) | 197 (190-201) | 42 (35-51) |
| 1 dose ChAdOx1-S, 1 dose BNT162b2 + mRNA-1273 booster | 23 | 84 (83-85) | 187 (184-196) | 48 (37-54) |
| 2 doses mRNA-1273 + BNT162b2 booster | 11 | 31 (28-41) | 182 (172-240) | 42 (32-51) |
| 2 doses BNT162b2 + mRNA-1273 booster | 11 | 33 (26-42) | 182 (156-226) | 42 (38-45) |
| 2 doses ChAdOx1-S + mRNA-1273 booster | 11 | 83 (81-84) | 188 (187-204) | 37 (33-43) |
| Other | 42 | 83 (42-85) | 192 (185-206) | 42 (37-61) |

Abbreviation: n: number of Participants, IQR: Interquartile Range

**Appendix Table 3. SARS-CoV-2 IgG antibody test results according to characteristics of HCW participants following COVID-19 vaccination (N=1517)**

| **Characteristic** | **IGG positive**  **(N=1482)** | **IGG negative/ borderline**  **(N=35)** |
| --- | --- | --- |
|  | **n (%)** | **n (%)** |
| Age groups in years |  |  |
| 16-29 | 264 (17.8%) | 3 (8.6%) |
| 30-39 | 411 (27.7%) | 14 (40.0%) |
| 40-49 | 348 (23.5%) | 12 (34.3%) |
| 50-59 | 333 (22.5%) | 5 (14.3%) |
| 60+ | 99 (6.7%) | 0 |
| Unknown | 27 (1.8%) | 1 (2.9%) |
| Gender |  |  |
| Female | 1085 (73.2%) | 27 (77.1%) |
| Male | 389 (26.2%) | 8 (22.9%) |
| Non-Binary | 4 (0.3%) | 0 |
| Unknown | 4 (0.3%) | 0 |
| Previous laboratory-confirmed SARS-CoV-2 infection |  |  |
| Yes, once | 210 (14.2%) | 4 (11.4%) |
| Yes, >1 reinfection | 11 (0.7%) | 0 |
| Time since previous laboratory confirmed SARS-CoV-2 infection |  |  |
| ≤ 3 months | 58 (3.9%) | 0 |
| > 3 months | 150 (10.1%) | 3 (8.6%) |
| unknown | 13 (0.9%) | 1 (2.9%) |
| No | 1253 (84.5%) | 31 (88.6%) |
| Unknown | 8 (0.5%) | 0 |
| Type of profession |  |  |
| Nurse | 454 (30.6%) | 13 (37.1%) |
| Physician | 259 (17.5%) | 3 (8.6%) |
| Other allied health professionals | 392 (26.5%) | 10 (28.6%) |
| Administration/other facility management | 356 (24.0%) | 8 (22.9%) |
| Unknown | 21 (1.4%) | 1 (2.9%) |
| Number of vaccine doses received |  |  |
| Three times vaccinated (plus evidence of past SARS-CoV-2 infection) | 79 (53.3%) | 0 |
| Three times vaccinated (no evidence of past SARS-CoV-2 infection) | 1121 (75.6%) | 1 (2.9%) |
| Two times vaccinated (plus evidence of past SARS-CoV-2 infection) | 98 (6.6%) | 0 |
| Two times vaccinated (no evidence of past SARS-CoV-2 infection) | 96 (6.5%) | 5 (14.3%) |
| One time vaccinated (plus evidence of past SARS-CoV-2 infection) | 32 (2.2%) | 0 |
| One time vaccinated (no evidence of past SARS-CoV-2 infection) | 15 (1.0%) | 4 (11.4%) |
| Not vaccinated (plus evidence of past SARS-CoV-2 infection) | 12 (0.8%) | 4 (11.4%) |
| Not vaccinated (no evidence of past SARS-CoV-2 infection) | 21 (1.4%) | 21 (60.0%) |
| Unknown | 8 (0.5%) | 0 |

Abbreviations: HCW: Health Care Worker, n: number of Participants, IgG: Immunoglobulin G

**Appendix Table 4. SARS-CoV-2 IgG antibody levels by COVID-19 vaccination status and timing of previous SARS-CoV-2** **infection (N=225)**

| **COVID-19 vaccination status and timing of previous SARS-CoV-2 infection** | **n (%)** | **Geometric Mean Titre¥ (95% CI)** |
| --- | --- | --- |
| Three times vaccinated (evidence of SARS-CoV-2 infection ≤3 months) | 16 (7.1%) | 2453 (1747-3442) |
| Three times vaccinated (evidence of SARS-CoV-2 infection >3 months) | 56 (24.9%) | 2261 (1923-2659) |
| Three times vaccinated (evidence of SARS-CoV-2 infection interval unknown) | 7 (3.1%) | 1407 (825-2399) |
| Two times vaccinated (evidence of SARS-CoV-2 infection ≤3 months) | 27 (12.0%) | 1964 (1341-2876) |
| Two times vaccinated (evidence of SARS-CoV-2 infection >3 months) | 66 (29.3%) | 1590 (1232-2053) |
| Two times vaccinated (evidence of SARS-CoV-2 infection interval unknown) | 5 (2.2%) | 3517 (1709-7239) |
| One time vaccinated (evidence of SARS-CoV-2 infection ≤3 months) | 7 (3.1%) | 869 (222-3390) |
| One time vaccinated (evidence of SARS-CoV-2 infection >3 months) | 25 (11.0%) | 1119 (741-1690) |
|  |  |  |
| One time vaccinated (evidence of SARS-CoV-2 infection interval unknown) | 0 | -- |
| Not vaccinated (evidence of SARS-CoV-2 infection ≤3 months) | 8 (3.5%) | 377 (116-1222) |
| Not vaccinated (evidence of SARS-CoV-2 infection >3 months) | 6 (2.7%) | 87 (9-831) |
| Not vaccinated (evidence of SARS-CoV-2 infection interval unknown) | 2 (0.9%) | 54 (--) |

Abbreviations: IgG: Immunoglobulin G, n – number of Participants, CI: Confidence Interval,

¥ SARS-CoV-2 Immunoglobulin G (IgG) antibody levels expressed as geometric mean titres (BAU/mL) with 95% confidence intervals (CIs)

**Appendix Figure 1. SARS-CoV-2 IgG antibody titre by type of COVID-19 vaccination among those vaccinated three times (N=1138)**


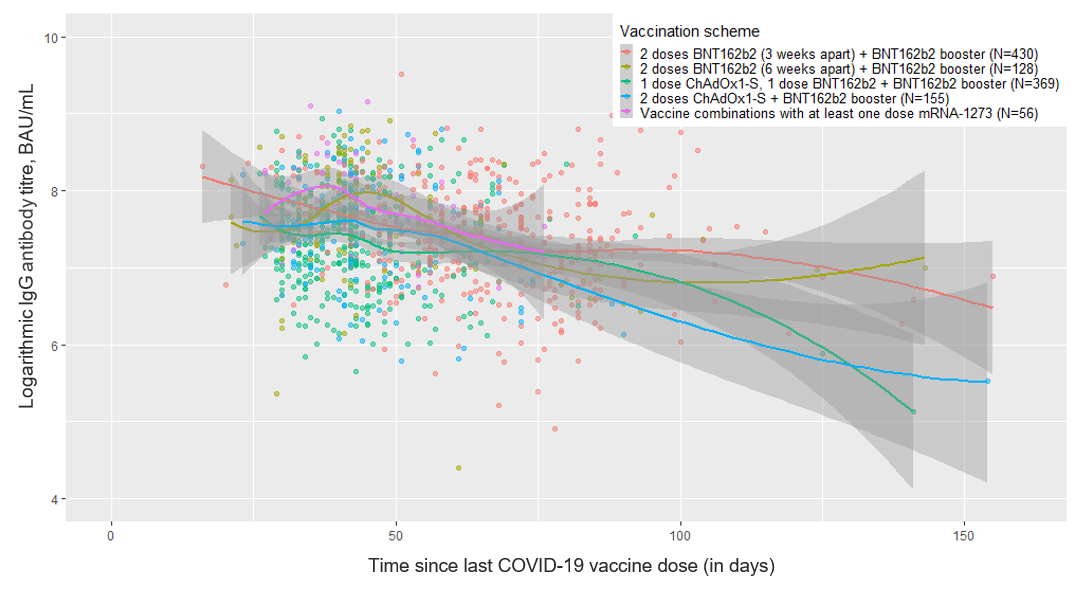


Abbreviation: IgG: Immunoglobulin G
